# Supplementary material for: Harnessing CD3 diversity to optimize CAR T cells
Source: Nat Immunol. 2023 Nov 6;24(12):2135–49. doi: 10.1038/s41590-023-01658-z (PMC10681901; doi:10.1038/s41590-023-01658-z)
Supplement: Supplementary file 1 — Supplementary Tables 1 and 2. [file 41590_2023_1658_MOESM1_ESM.pdf]

nature immunology

Article https://doi.org/10.1038/s41590-023-01658-z

Harnessing CD3 diversity to optimize CAR T cells

In the format provided by the authors and unedited

nature immunology

Article https://doi.org/10.1038/s41590-023-01658-z

Harnessing CD3 diversity to optimize CAR T cells

In the format provided by the authors and unedited

Supplementary Table 1. Antibodies used for mass cytometry (CyTOF)

Channel	Antigen	Supplier	Clone	Cat#
89Y	CD45	fluidigm	HI30	3089003B
104Pd	β2m-Barcode	Biolegend	2M2	316302
105Pd	β2m-Barcode	Biolegend	2M2	316302
106Cd	β2m-Barcode	Biolegend	2M2	316302
108Pd	β2m-Barcode	Biolegend	2M2	316302
110Pd	β2m-Barcode	Biolegend	2M2	316302
111 Cd	CD4	Biolegend	RPA-T4	300502
112 Cd	CD3	Biolegend	UCHT1	300402
113 Cd	CD39	Biolegend	A1	328202
114 Cd	IRF4	Invitrogen	3E4	14-9858-82
115 In	CD57	Invitrogen	TB01	16-0577-85
116 Cd	CD8	Biolegend	RPA-T8	301002
139 La	MM-DOTA			
140 etc	Beads			
141 Pr	CD19	Biolegend	HIB19	302202
142 Nd	IFN-γ	Biolegend	B27	506502
143 Nd	HIF1α	Abcam	EP1215Y	ab210073
144 Nd	CTLA-4	BD	BNI3	555850
145 Nd	TNF-α	Invitrogen	MAb11	14-7349-85
146 Nd	Ki-67	BD	B56	556003
147 Sm	CD45RA	Biolegend	HI100	304143
148 Nd	ACC	Cell Signaling Technology	C83B10	3676BF
149 Sm	GLS1	Cell Signaling Technology	E4T9Q	49363BF
150 Nd	CD127	Biolegend	A019D5	351302
151 Eu	Flag-Tag (anti-DYKDDDDK)	BioLegend	L5	637301
152 Sm	IL-2	Invitrogen	MQ1-17H12	14-7029-85
153 Eu	CPT1α	Cell Signaling Technology	D3B3	12252BF
154 Sm	XCL-1	R&D	109001	MAB6951
155 Gd	CD27	Biolegend	O323	302802
156 Gd	TOMM20	Abcam	ERP15581-54	ab232589
157 Gd	FOXO1	Biolegend	2F8B08	658102
158 Gd	PD-1	Biolegend	EH12.2H7	329902
159 Tb	CCR7	Biolegend	G043H7	353255
160 Gd	Tbet	Biolegend	4B10	644802
161 Dy	CD28	Biolegend	CD28.2	302902
162 Dy	Tim-3	BioLegend	F38-2E2	345002
163 Dy	TCF-1	Biolegend	7F11A10	615702

164 Dy	CytC	Biolegend	6H2.B4	612302
165 Ho	Eomes	Invitrogen	WD1928	14-4877-82
166 Er	Bodipy	Invitrogen	poly	A5770
167 Er	CD38	Biolegend	HIT2	303502
168 Er	TOX	Milteny	REA473	130-095-212
169 Tm	TIGIT	Invitrogen	MBSA43	16-9500-82
170 Er	LAG-3	Biolegend	11C3C65	369302
171 YB	2B4	Biolegend	C1.7	329502
172 Yb	CD71	Invitrogen	OKT9	14-0719-82
173 Yb	CCL3/4	R&D	93342	MAB2701
174 Yb	IL-10	Biolegend	JES3-9D7	501402
175 Lu	GLUT1	Abcam	EPR3915	ab252403
176 Yb	CD36	Biolegend	5-271	336202
191/193	Iridium			
194Pt	Perforin	abcam	B-D48	ab47225
198 Pt	β2m-Barcode			
209 Bi	CD16	fluidigm	3G8	3165001C

Supplementary Table 2: Antibodies used for Flow Cytometry

Conjugate	Antigen	Supplier	Clone	Cat#
	Strep-Tag (NWSHPQFEK)	Genscript	5A9F9	A01736
APC	Flag-Tag (DYKDDDDK)	BioLegend	L5	637308
Biotin	mouse IgG	Invitrogen		31803
Biotin	human PD-1	BioLegend	EH12.2H7	329934
PECy7	human Tim-3	BioLegend	F382E2	345014
Alexa 647	human LAG-3	BioLegend	11C3C65	369304
PE	human CD25	BioLegend	BC96	302606
PECy7	human CD137	Invitrogen	4B4-1	25-1379-42
APC	human CD69	Invitrogen	CH/4	MHCD6905
PE	human CD8	Beckman Coulter	B9.11	IM0452U
APC	human CD8	Beckman Coulter	B9.11	IM2469
PECy7	human CD4	eBioscience	RPA-T4	25-0049-42
PE	human CD107a	Biolegend	H4A3	328608
PECy7	human CD4	eBioscience	14D3	25-1529-42
PECy7	human TCF1/TCF7	Cell Signaling Technology	C63D9	90511
PECy7	human CD45RA	Biolegend	HI100	304126
APC	human CD27	BD Pharmingen	MT271	561400
BV421	human CD3	BioLegend	UCHT1	300434
Alexa 488	human CD3	BioLegend	UCHT1	300415
APC	Streptavidin	BioLegend		405207
PECy7	Streptavidin	Invitrogen		SA1012
eFluor 450	Streptavidin	Invitrogen		48-4317-82
