# Supplementary material for: Harnessing CD3 diversity to optimize CAR T cells
Source: Nat Immunol. 2023 Nov 6;24(12):2135–49. doi: 10.1038/s41590-023-01658-z (PMC10681901; doi:10.1038/s41590-023-01658-z)
Supplement: Supplementary file 2 — Reporting Summary [file 41590_2023_1658_MOESM2_ESM.pdf]

nature portfolio

Corresponding author(s): Prof Dr. Susana Minguet

Last updated by author(s): 31/07/2023

Reporting Summary

Nature Portfolio wishes to improve the reproducibility of the work that we publish. This form provides structure for consistency and transparency in reporting. For further information on Nature Portfolio policies, see our Editorial Policies and the Editorial Policy Checklist.

Statistics

For all statistical analyses, confirm that the following items are present in the figure legend, table legend, main text, or Methods section.

n/a	Confirmed	
☐	☒	The exact sample size (*n*) for each experimental group/condition, given as a discrete number and unit of measurement
☐	☒	A statement on whether measurements were taken from distinct samples or whether the same sample was measured repeatedly
☐	☒	The statistical test(s) used AND whether they are one- or two-sided<br>*Only common tests should be described solely by name; describe more complex techniques in the Methods section.*
☒	☐	A description of all covariates tested
☒	☐	A description of any assumptions or corrections, such as tests of normality and adjustment for multiple comparisons
☐	☒	A full description of the statistical parameters including central tendency (e.g. means) or other basic estimates (e.g. regression coefficient) AND variation (e.g. standard deviation) or associated estimates of uncertainty (e.g. confidence intervals)
☐	☒	For null hypothesis testing, the test statistic (e.g. *F*, *t*, *r*) with confidence intervals, effect sizes, degrees of freedom and *P* value noted<br>*Give P values as exact values whenever suitable.*
☒	☐	For Bayesian analysis, information on the choice of priors and Markov chain Monte Carlo settings
☒	☐	For hierarchical and complex designs, identification of the appropriate level for tests and full reporting of outcomes
☐	☒	Estimates of effect sizes (e.g. Cohen's *d*, Pearson's *r*), indicating how they were calculated

Our web collection on statistics for biologists contains articles on many of the points above.

Software and code

Policy information about availability of computer code

Data collection	Cytokines were measured by ELISA with Thermo Scientific MultiScan.<br>For SLB experiments an iXon Ultra 897 EMCCD camera (Oxford Instruments) was used for data recording.<br>Bioluminescence-based cytotoxicity assay: Luminometer (Tecan infinity M200 Pro).<br>Flow cytometry: Gallios FACS, Beckman Coulter and Attune NxT FACS, Thermo Fisher Scientific.<br>Bioluminescence imaging: IVIS SpectrumCT System.<br>CyTOF (Helios)

Data analysis	Immunobotting: Quantification of the band intensities was performed with the Image Lab Software from BioRad after chemiluminescence detection in an Image Quant LAS 4000 Mini (GE Healthcare).<br>Flow cytometry analysis: Flowjo Software V10<br>Statistics: GraphPad Prism 9<br>SLB experiments were analysed with an inhouse custom-built Matlab software was used to track cells in each frame using a particle tracking algorithm.<br>Gene expression data was analysed nSolver 4.0 software. Downstream bioinformatic analysis was performed with R (4.2.1). Briefly, the differentially regulated genes were identified using the limma R package, with a paired design (donor-based). Adjusted p value (Benjamini Hochberg) below 0.05 was considered as significant. The Generally Applicable Gene-set Enrichment (GAGE) R package was used to identified regulated gene-sets among the whole MSigDB repository and the NanoString panel gene-sets.<br>Cytof: Normalizer (https://www.github.com/nolanlab/bead- normalization/releases), FlowJo (v10), Omiq (https://www.r-project.org) and R (https://www.r-project.org/).

For manuscripts utilizing custom algorithms or software that are central to the research but not yet described in published literature, software must be made available to editors and reviewers. We strongly encourage code deposition in a community repository (e.g. GitHub). See the Nature Portfolio guidelines for submitting code & software for further information.

Data

Policy information about availability of data

All manuscripts must include a data availability statement. This statement should provide the following information, where applicable:

- Accession codes, unique identifiers, or web links for publicly available datasets
- A description of any restrictions on data availability
- For clinical datasets or third party data, please ensure that the statement adheres to our policy

Transcriptome data have been deposited in the Gene Expression Omnibus under the accession number GSE243226. Source Data are provided with this paper. All other data that support the findings of this study are present in the article or are available from the corresponding author upon request.

Human research participants

Policy information about studies involving human research participants and Sex and Gender in Research.

Reporting on sex and gender	*Use the terms sex (biological attribute) and gender (shaped by social and cultural circumstances) carefully in order to avoid confusing both terms. Indicate if findings apply to only one sex or gender; describe whether sex and gender were considered in study design whether sex and/or gender was determined based on self-reporting or assigned and methods used. Provide in the source data disaggregated sex and gender data where this information has been collected, and consent has been obtained for sharing of individual-level data; provide overall numbers in this Reporting Summary. Please state if this information has not been collected. Report sex- and gender-based analyses where performed, justify reasons for lack of sex- and gender-based analysis.*
Population characteristics	*Describe the covariate-relevant population characteristics of the human research participants (e.g. age, genotypic information, past and current diagnosis and treatment categories). If you filled out the behavioural & social sciences study design questions and have nothing to add here, write "See above."*
Recruitment	*Describe how participants were recruited. Outline any potential self-selection bias or other biases that may be present and how these are likely to impact results.*
Ethics oversight	*Identify the organization(s) that approved the study protocol.*

Note that full information on the approval of the study protocol must also be provided in the manuscript.

Field-specific reporting

Please select the one below that is the best fit for your research. If you are not sure, read the appropriate sections before making your selection.

☒ Life sciences ☐ Behavioural & social sciences ☐ Ecological, evolutionary & environmental sciences

For a reference copy of the document with all sections, see nature.com/documents/nr-reporting-summary-flat.pdf

Life sciences study design

All studies must disclose on these points even when the disclosure is negative.

Sample size	For animal studies, the recommendations from Prof. Dr. Hauschke (Institute for Med. Biometry and Med. Informatics, University of Freiburg) were followed. These recommendations were enclosed in the animal protocol following the German animal protection law and with permission from the responsible local authorities. Briefly, The sample size was calculated using the following parameters: 1.06 effect size, 5% significance level, 80% power, and 1.06 standard deviation.

	For ex-vivo experiments, at least two experiments were performed to estimate the standard deviation and the effect size. These estimations were applied to the Lehr's formula to estimate the sample size needed.
Data exclusions	Animals which do not fill up the health-criteria described in the animal protocol that at the moment of the analysis were excluded. The criteria were therefore pre-established at the animal protocol. Failed Experiments due to technical issues were excluded.
Replication	Experiments were replicate at least twice and statistics were done to verify reproducibility. All attends of replications were sucessful.
Randomization	Allocation was ramdom
Blinding	Blinding was not done (impractical due to the difficulties to control all variables without implying mistakes)

Reporting for specific materials, systems and methods

We require information from authors about some types of materials, experimental systems and methods used in many studies. Here, indicate whether each material, system or method listed is relevant to your study. If you are not sure if a list item applies to your research, read the appropriate section before selecting a response.

Materials & experimental systems

n/a	Involved in the study
☐	☒ Antibodies
☐	☒ Eukaryotic cell lines
☒	☐ Palaeontology and archaeology
☐	☒ Animals and other organisms
☒	☐ Clinical data
☒	☐ Dual use research of concern

Methods

n/a	Involved in the study
☒	☐ ChIP-seq
☐	☒ Flow cytometry
☒	☐ MRI-based neuroimaging

Antibodies

Antibodies used

Antibodies used for mass cytometry (CyTOF):
Channel Antigen Supplier Clone Cat#
89Y CD45 fluidigm HI30 3089003B
104Pd β2m-Barcode Biolegend 2M2 316302
105Pd β2m-Barcode Biolegend 2M2 316302
106Cd β2m-Barcode Biolegend 2M2 316302
108Pd β2m-Barcode Biolegend 2M2 316302
110Pd β2m-Barcode Biolegend 2M2 316302
111 Cd CD4 Biolegend RPA-T4 300502
112 Cd CD3 Biolegend UCHT1 300402
113 Cd CD39 Biolegend A1 328202
114 Cd IRF4 Invitrogen 3E4 14-9858-82
115 In CD57 Invitrogen TB01 16-0577-85
116 Cd CD8 Biolegend RPA-T8 301002
139 La MM-DOTA
140 etc Beads
141 Pr CD19 Biolegend HIB19 302202
142 Nd IFN-? Biolegend B27 506502
143 Nd HIF1? Abcam EP1215Y ab210073
144 Nd CTLA-4 BD BNI3 555850
145 Nd TNF-? Invitrogen MAb11 14-7349-85
146 Nd Ki-67 BD B56 556003
147 Sm CD45RA Biolegend HI100 304143
148 Nd ACC Cell Signaling Technology C83B10 3676BF
149 Sm GLS1 Cell Signaling Technology E4T9Q 49363BF
150 Nd CD127 Biolegend A019D5 351302
151 Eu Flag-Tag (anti-DYKDDDDK) BioLegend L5 637301
152 Sm IL-2 Invitrogen MQ1-17H12 14-7029-85
153 Eu CPT1? Cell Signaling Technology D3B3 12252BF
154 Sm XCL-1 R&D 109001 MAB6951
155 Gd CD27 Biolegend O323 302802
156 Gd TOMM20 Abcam ERP15581-54 ab232589
157 Gd FOXO1 Biolegend 2F8B08 658102
158 Gd PD-1 Biolegend EH12.2H7 329902
159 Tb CCR7 Biolegend G043H7 353255
160 Gd Tbet Biolegend 4B10 644802
161 Dy CD28 Biolegend CD28.2 302902
162 Dy Tim-3 BioLegend F38-2E2 345002
163 Dy TCF-1 Biolegend 7F11A10 615702

164 Dy CytC Biolegend 6H2.B4 612302
165 Ho Eomes Invitrogen WD1928 14-4877-82
166 Er Bodipy Invitrogen poly A5770
167 Er CD38 Biolegend HIT2 303502
168 Er TOX Milteny REA473 130-095-212
169 Tm TIGIT Invitrogen MBSA43 16-9500-82
170 Er LAG-3 Biolegend 11C3C65 369302
171 YB 2B4 Biolegend C1.7 329502
172 Yb CD71 Invitrogen OKT9 14-0719-82
173 Yb CCL3/4 R&D 93342 MAB2701
174 Yb IL-10 Biolegend JES3-9D7 501402
175 Lu GLUT1 Abcam EPR3915 ab252403
176 Yb CD36 Biolegend 5-271 336202
191/193 Iridium
194Pt Perforin abcam B-D48 ab47225
198 Pt β2m-Barcode
209 Bi CD16 fluidigm 3G8 3165001C
Antibodies used for Flow Cytometry:
Conjugate Antigen Supplier Clone Cat#
Biotin Strep-Tag (NWSHPQFEK) Genscript 5A9F9 A01736
APC Flag-Tag (DYKDDDDK) BioLegend L5 637308
Biotin mouse IgG Invitrogen 31803
Biotin human PD-1 BioLegend EH12.2H7 329934
PECy7 human Tim-3 BioLegend F382E2 345014
Alexa 647 human LAG-3 BioLegend 11C3C65 369304
PE human CD25 BioLegend BC96 302606
PECy7 human CD137 Invitrogen 4B4-1 25-1379-42
APC human CD69 Invitrogen CH/4 MHCD6905
PE human CD8 Beckman Coulter B9.11 IM0452U
APC human CD8 Beckman Coulter B9.11 IM2469
PECy7 human CD4 eBioscience RPA-T4 25-0049-42
PE human CD107a Biolegend H4A3 328608
PECy7 human CD4 eBioscience 14D3 25-1529-42
PECy7 human TCF1/TCF7 Cell Signaling Technology C63D9 90511
PECy7 human CD45RA Biolegend HI100 304126
APC human CD27 BD Pharmingen MT271 561400
BV421 human CD3 BioLegend UCHT1 300434
Alexa 488 human CD3 BioLegend UCHT1 300415
APC Streptavidin BioLegend 405207
PECy7 Streptavidin Invitrogen SA1012
eFluor 450 Streptavidin Invitrogen 48-4317-82

anti-human CD3 (UCHT-1, from J. Bluestone, UC San Francisco, USA) and anti-human CD28 (CD28.2, BioLegend) were used. Primary human T cells were grown with human IL-2 (Perprotech). For transduction, Proteamine Sulfate salt from herring (Sigma, Life Sciences) was used. For immunoprecipitation the THE™ NWSHPQFEK Tag Antibody (5A9F9, Genscript) or the biotin-coupled anti-mouse IgG (Fab´)2 biotin (AB_228311, Thermo Fisher) and the anti-human SH-PTP1 (D11 or C19, Santa Cruz Biotechnology) were used. Precision Count Beads (BioLegend) were employed to count the cells in the rechallenge and CyTOF experiments. For the rapid Expansion Protocol, anti-human CD3� (OKT-3) was utilized.

Validation

All antibodies were purchased from manufactures that indicate validation in the data sheet. Antibodies for Western blot were validated using cells lacking the protein of interest and by protein size upon SDS-PAGE. Antibodies used in flow and mass cytometry were further validate using cell mixtures containing cells expressing or lacking the protein recognized by the antibody.

Eukaryotic cell lines

Policy information about cell lines and Sex and Gender in Research

Cell line source(s)

HEK293T (CRL-1573) and Jurkat (TIB-152) were obtained from ATCC. 2B4 cells were obtained from Balbino Alarcon (CBM-SO, Spain). Nalm6 cells were obtained from TCR2 therapeutics.

Authentication

Cells were authenticated by expression of surface markers by Flow cytometry.

Mycoplasma contamination

All cell lines were tested negative for Mycoplasm contamination.

Commonly misidentified lines
(See ICLAC register)

No cell line listed by ICLAC was used.

Animals and other research organisms

Policy information about studies involving animals; ARRIVE guidelines recommended for reporting animal research, and Sex and Gender in Research

Laboratory animals

Mice were used in this study. Rag2-γ- (Rag2tm1.1Flv Il2rgtm1.1Flv) were purchased from Jackson Laboratory, bred and kept at the

Laboratory animals	Center for Experimental Models and Transgenic Service, Freiburg, under specific pathogen-free conditions. Both adult (older than 8 weeks) females and males were used.
Wild animals	The study did not involved wild animals
Reporting on sex	The sex was not considered for the study design
Field-collected samples	The study did not involved field-collected samples
Ethics oversight	All animal protocols (G18/03) were performed according to the German animal protection law with permission from the Veterinär und Lebensmittelüberwachungsbehörde Freiburg.

Note that full information on the approval of the study protocol must also be provided in the manuscript.

Flow Cytometry

Plots

Confirm that:

☒ The axis labels state the marker and fluorochrome used (e.g. CD4-FITC).

☒ The axis scales are clearly visible. Include numbers along axes only for bottom left plot of group (a 'group' is an analysis of identical markers).

☒ All plots are contour plots with outliers or pseudocolor plots.

☒ A numerical value for number of cells or percentage (with statistics) is provided.

Methodology

Sample preparation	For extracellular staining, cells were collected, washed once with FACS Buffer (PBS + 2% FBS), and stained for 15 minutes at 4°C in the darkness. Then, cells were washed twice with FACS buffer and acquisition was performed. Intracellular staining has been performed using eBioscience FOXP3/Transcription Factor Staining Buffer Set (Invitrogen). Briefly, cells were washed with FACS buffer and subsequently resuspended in 1xFixation/Permeabilization solution and incubated for 30 minutes at 4°C. After fixation and permeabilization, cells were washed twice with 1xPermeabilization buffer and intracellular staining of TCF1 has been performed at RT for 30 minutes. After two washing steps with 1xPermeabilization buffer cells have been resuspended in FACS buffer.
Instrument	Gallios FACS, Beckman Coulter or in Attune® NxT Acoustic Focusing Cytometer, Invitrogen.
Software	FlowJo Software
Cell population abundance	Upon sorting, purifty <95% was obteined,
Gating strategy	Gating strategy is now depicted in Extended Data Fig. 8

☒ Tick this box to confirm that a figure exemplifying the gating strategy is provided in the Supplementary Information.
