# Supplementary figures and images for: Harnessing CD3 diversity to optimize CAR T cells
Source: Nat Immunol. 2023 Nov 6;24(12):2135–49. doi: 10.1038/s41590-023-01658-z (PMC10681901; doi:10.1038/s41590-023-01658-z)

L (M6547)

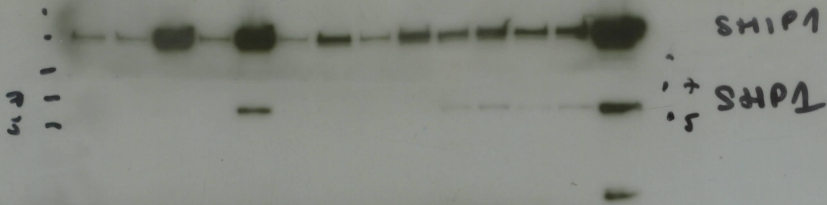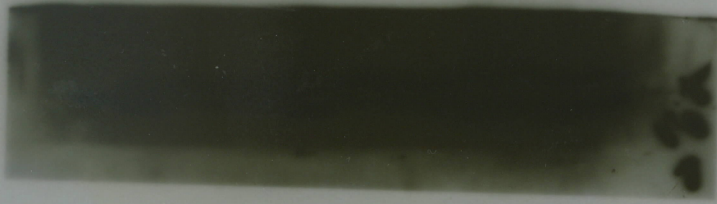

Aud-HRP

18.3.17

10% gel

Supplement: Supplementary file 12 — Blots_Figure_8d. [file 41590_2023_1658_MOESM12_ESM.pdf]

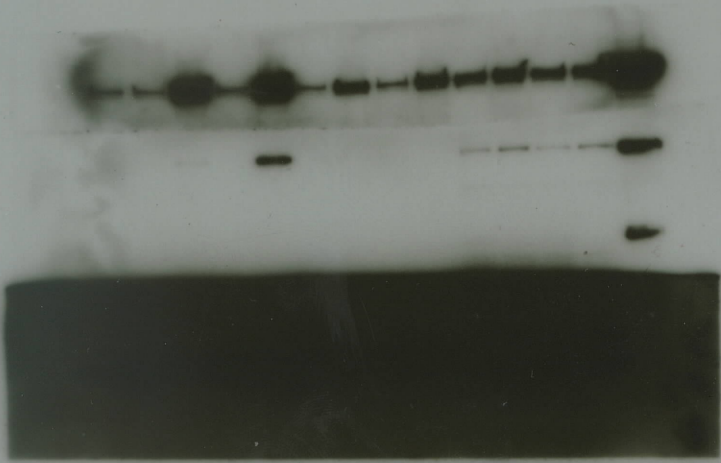

SHIP  
SHP

☆

pept.

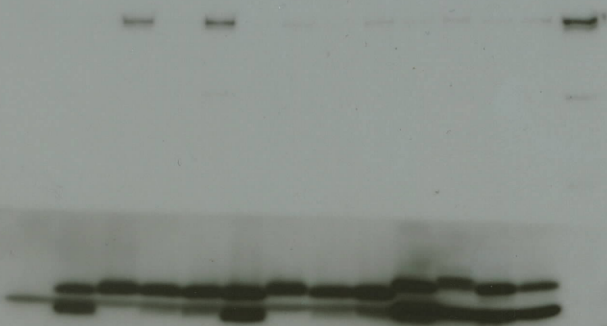

☆

pept.

18.3.17

Supplement: Supplementary file 13 — Blots_Figure_8d. [file 41590_2023_1658_MOESM13_ESM.pdf]

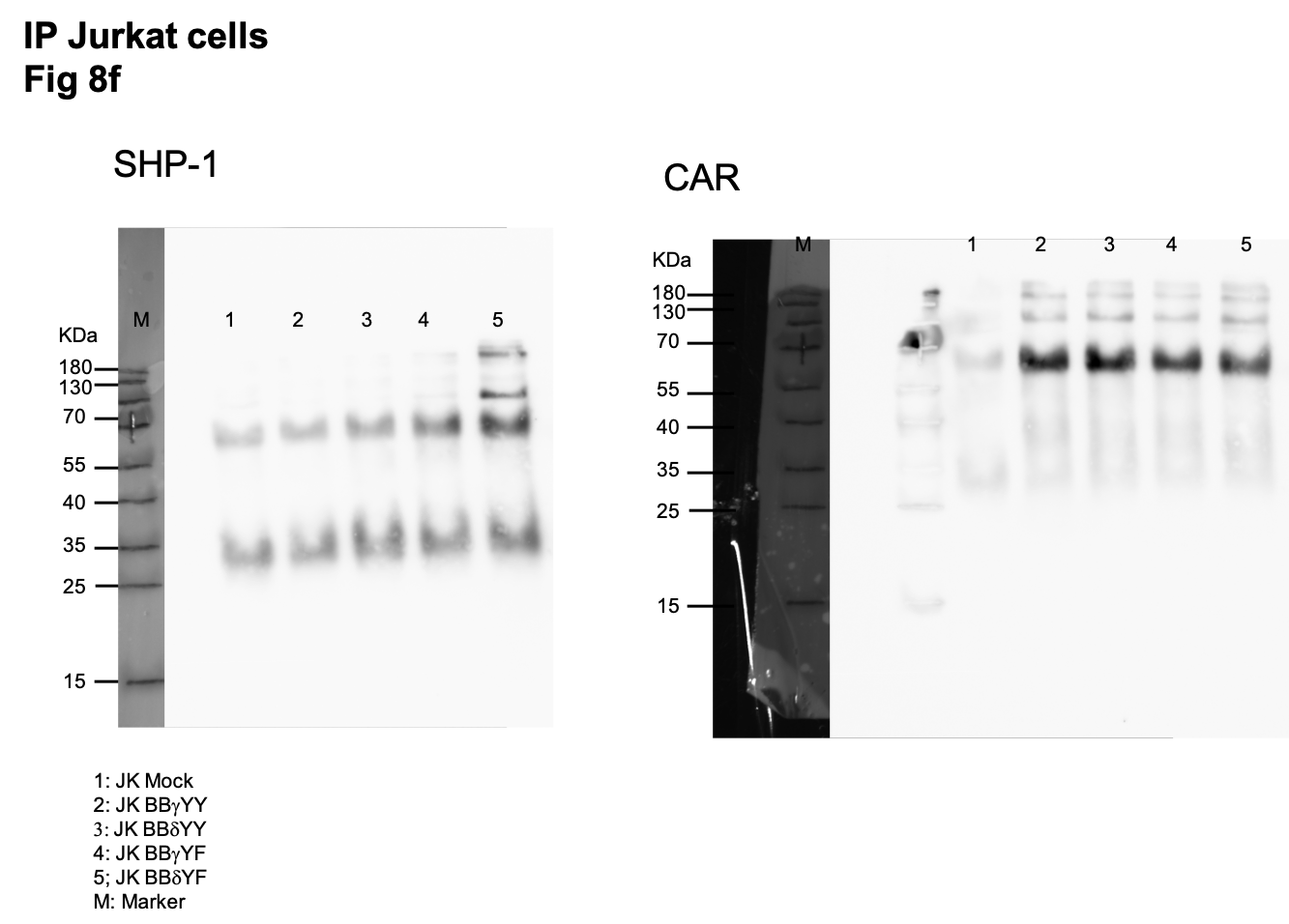

Supplement: Supplementary file 14 — Blots_Figure_8f marker. [file 41590_2023_1658_MOESM14_ESM.tif]

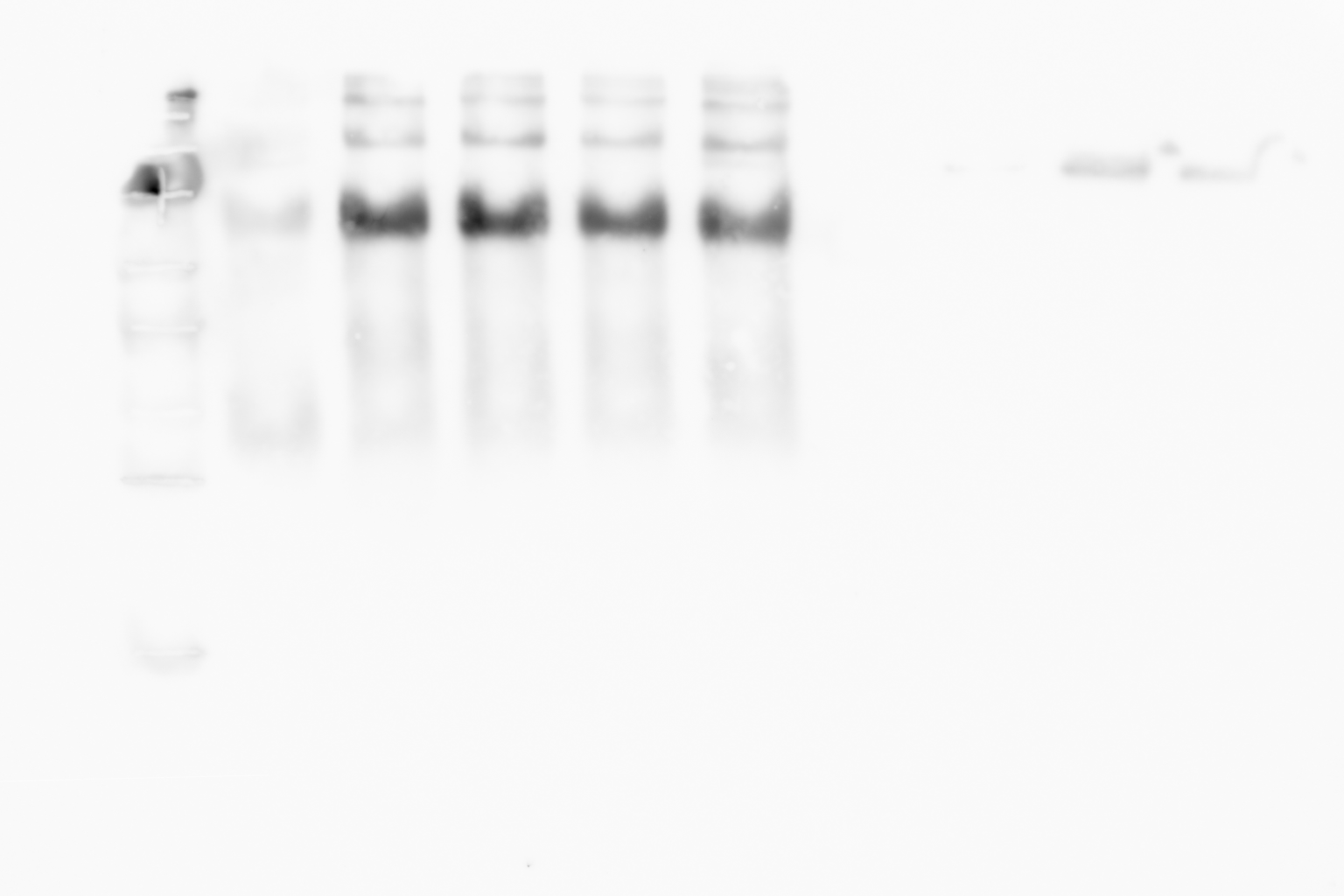

Supplement: Supplementary file 15 — Uncropped blot Fig. 8f, CAR development. [file 41590_2023_1658_MOESM15_ESM.tif]

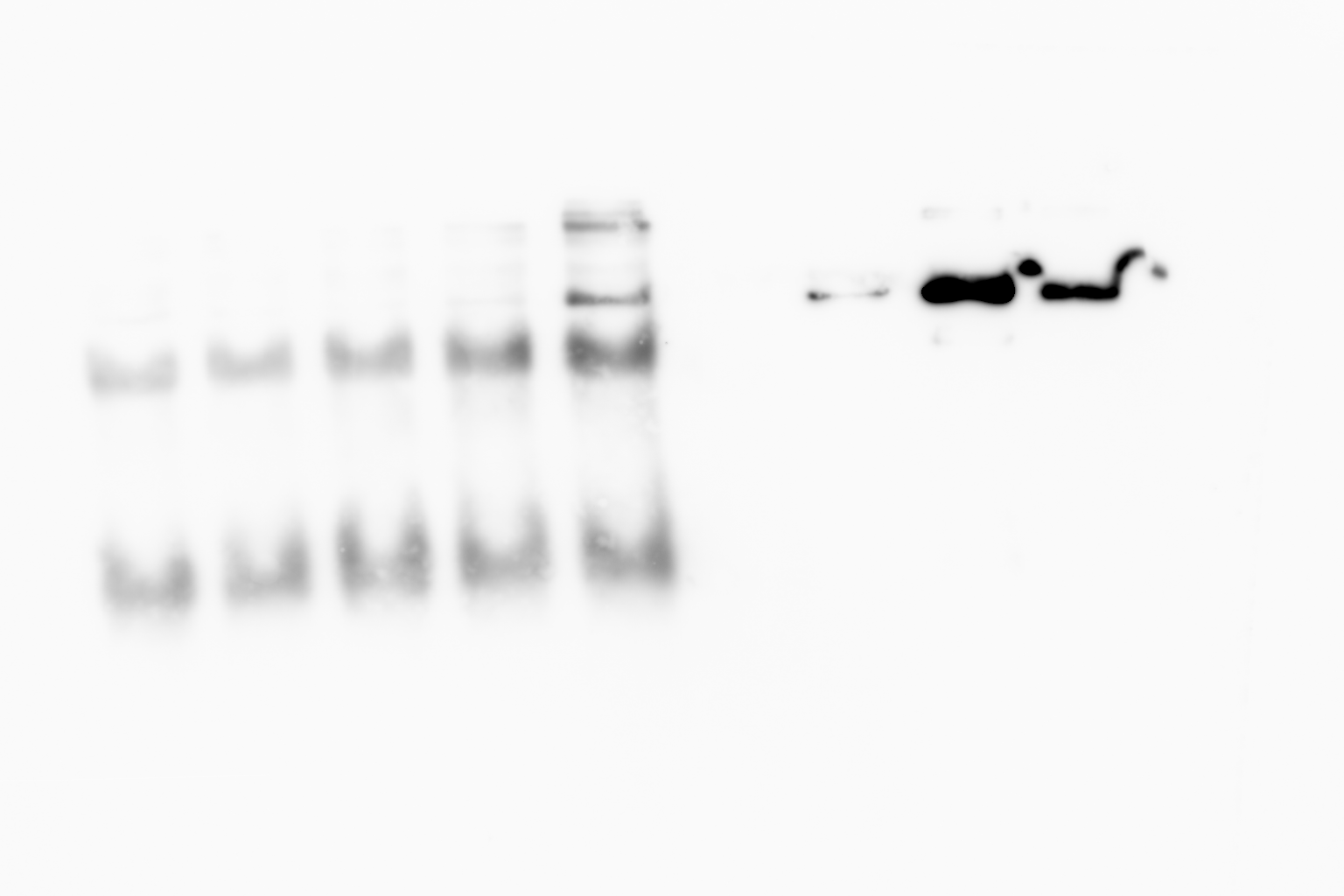

Supplement: Supplementary file 16 — Uncropped blot Fig. 8f, SHP-1 development. [file 41590_2023_1658_MOESM16_ESM.tif]

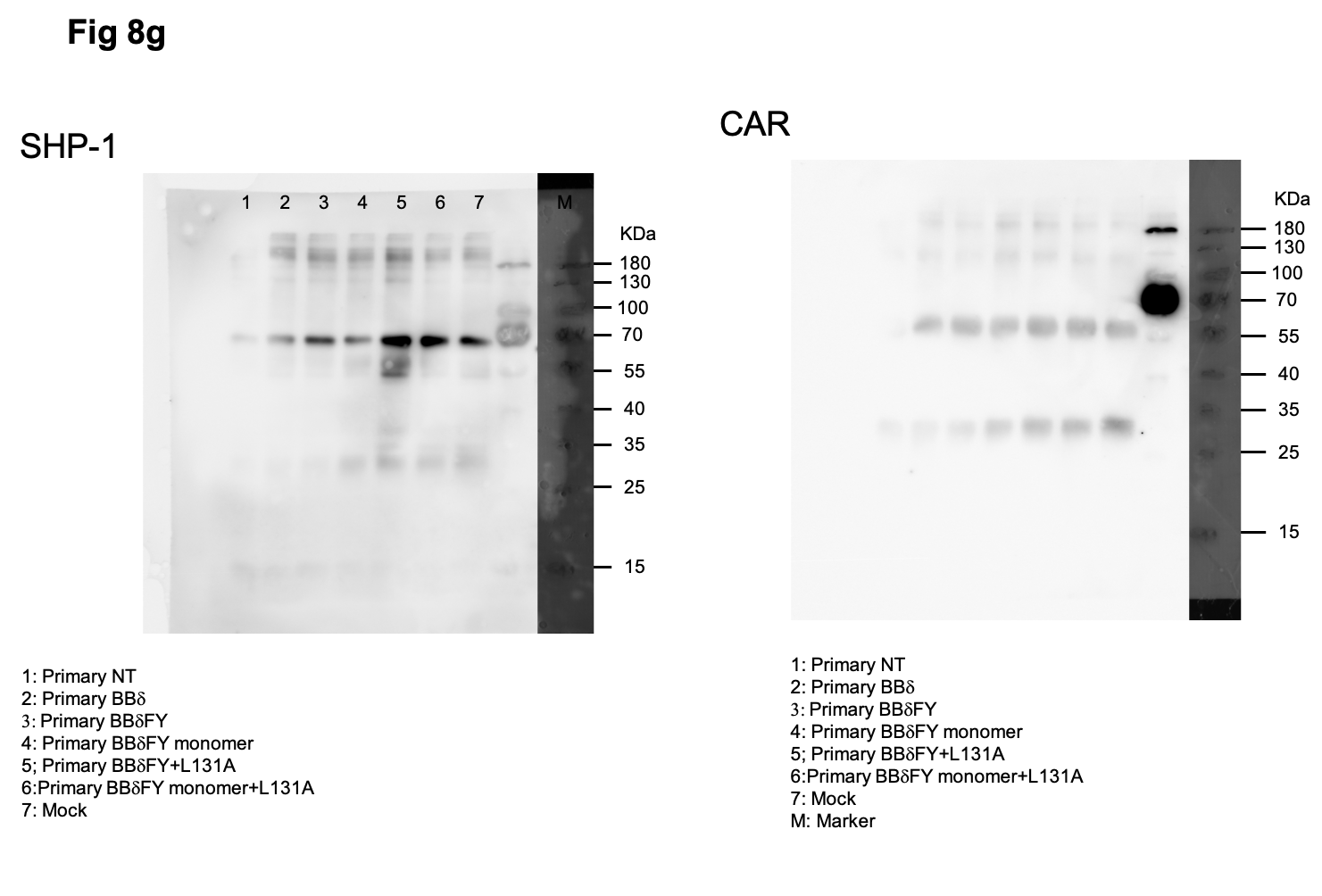

Supplement: Supplementary file 17 — Blots_Figure_8f marker. [file 41590_2023_1658_MOESM17_ESM.tif]

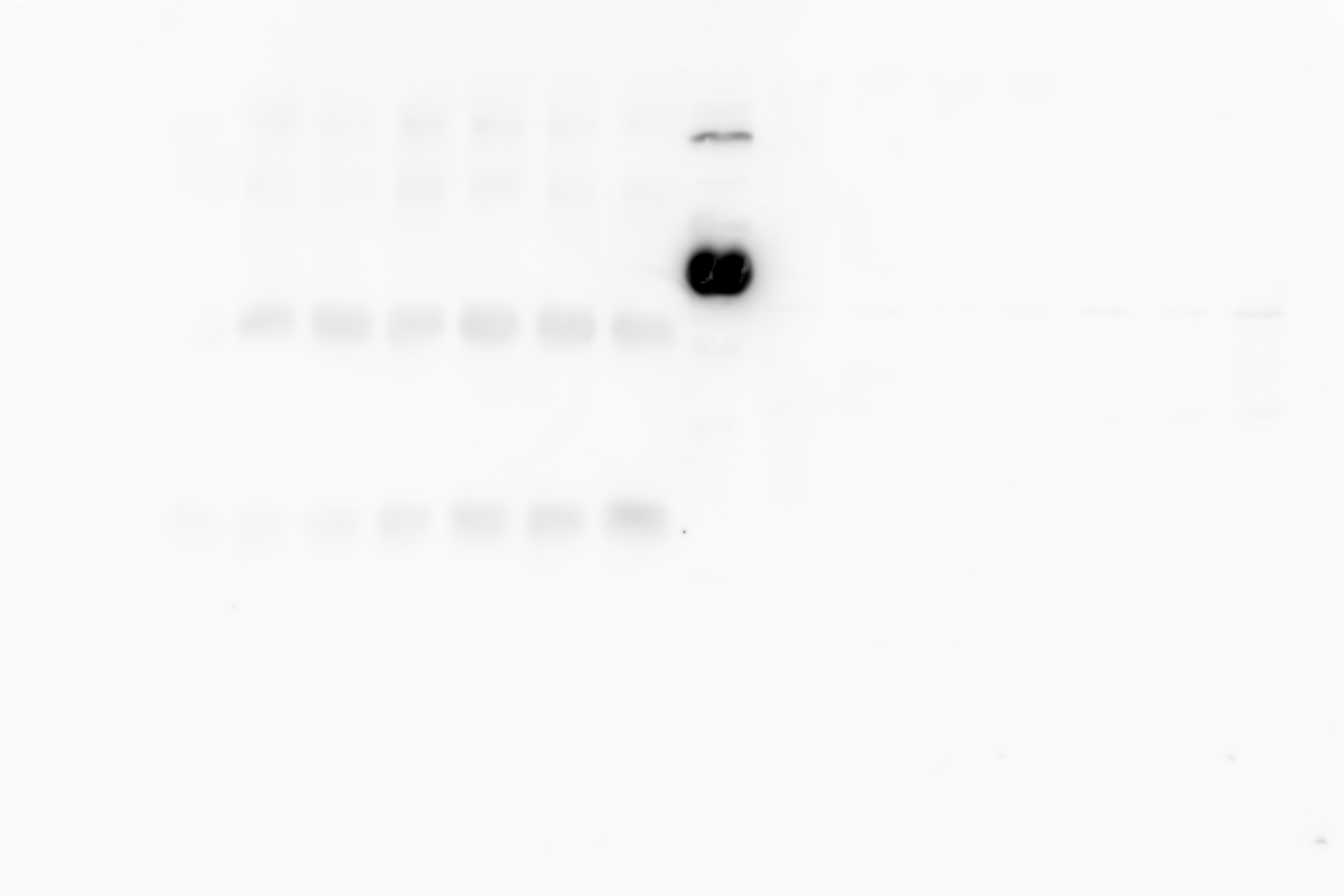

Supplement: Supplementary file 18 — Uncropped blot Fig. 8g, CAR development. [file 41590_2023_1658_MOESM18_ESM.tif]

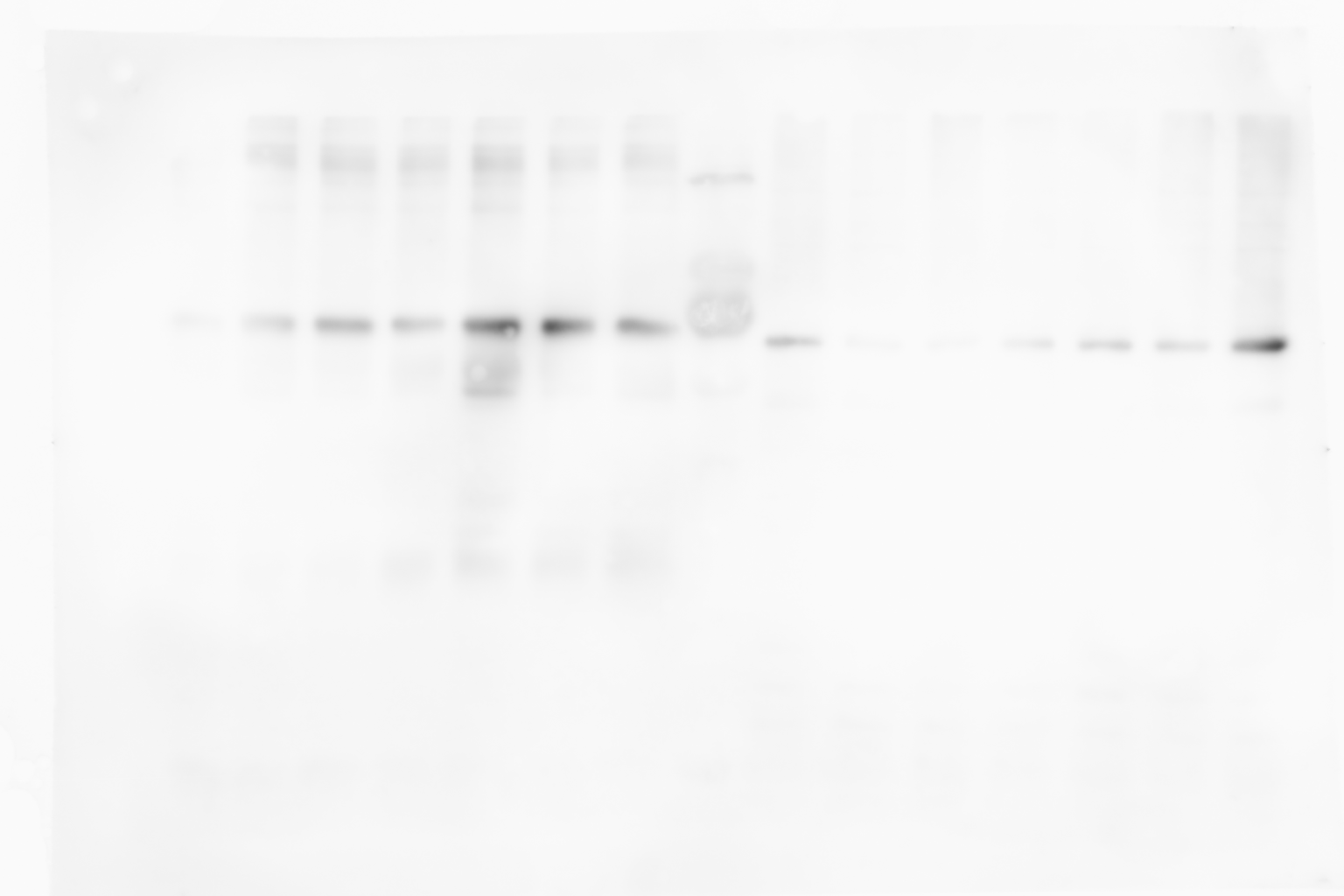

Supplement: Supplementary file 19 — Uncropped blot Fig. 8g, SHP-1 development. [file 41590_2023_1658_MOESM19_ESM.tif]
